# Elimination of Antibiotic Resistant Surgical Implant Biofilms Using an Engineered Cationic Amphipathic Peptide WLBU2

**DOI:** 10.1038/s41598-017-17780-6

**Published:** 2017-12-22

**Authors:** Jonathan B. Mandell, Berthony Deslouches, Ronald C. Montelaro, Robert M. Q. Shanks, Yohei Doi, Kenneth L. Urish

**Affiliations:** 1 Arthritis and Arthroplasty Design Group, Department of Orthopaedic Surgery, University of Pittsburgh, Pittsburgh, PA USA; 2 Department of Microbiology and Molecular Genetics, University of Pittsburgh, Pittsburgh, PA USA; 3 Department of Ophthalmology, University of Pittsburgh, Pittsburgh, PA USA; 4 Division of Infectious Diseases, School of Medicine, University of Pittsburgh, Pittsburgh, PA USA; 5 The Magee Bone and Joint Center, University of Pittsburgh Medical Center, Pittsburgh, PA USA; 6 Clinical & Translational Science Institute, University of Pittsburgh, Pittsburgh, PA USA; 7 Department of Bioengineering, University of Pittsburgh, Pittsburgh, PA USA; 8 Department of Biomedical Engineering, Carnegie Mellon University, Pittsburgh, PA USA

## Abstract

Antibiotics are unable to remove biofilms from surgical implants. This high antibiotic tolerance is related to bacterial persisters, a sub-population of bacteria phenotypically tolerant to antibiotics secondary to a reduced metabolic state. WLBU2 is an engineered cationic amphipathic peptide designed to maximize antimicrobial activity with minimal mammalian cell toxicity. The objective of this study was to test the ability of WLBU2 to remove *Staphylococcus aureus* surgical implant biofilms. WLBU2 effectively treated *S. aureus* biofilms formed by a variety of clinical MSSA and MRSA strains and created culture-negative implants in the *in vitro* biofilm model. Blocking bacterial metabolism by inhibiting oxidative phosphorylation did not affect WLBU2 killing compared to decreased killing by cefazolin. In the surgical implant infection animal model, WLBU2 decreased biofilm mass as compared to control, untreated samples. WLBU2 could rapidly eliminate implants *in vitro* and had sufficient efficacy *in vivo* with minimal systemic toxicity.

## Introduction

Infection remains an enormous clinical challenge in the field of surgery, and greatly increases the risk of morbidity and mortality for the patient. Total knee arthroplasty or knee replacement surgery provides an example of this dilemma. Given its success and cost feasibility, total knee arthroplasty has become one of the largest major surgical procedures by volume in the United States^1,2^. However, infection remains the most serious and costly reason for total knee arthroplasty failure^3,4^. An infected total knee arthroplasty, termed periprosthetic joint infection, is a devastating diagnosis. Treatment options are few and require repeat surgical intervention with long-term antibiotic regimen^5^. Five-year mortality for periprosthetic joint infection is approximately 25%, higher than three of the most common cancers of melanoma, breast, and prostate^6,7^. The most common organism in surgical site infection and periprosthetic joint infection is *Staphylococcus aureus* ^8,9^. First-line treatment for these infections include first generation cephalosporins such as cefazolin for methicillin-susceptible strains and vancomycin for methicillin-resistant strains^5^.

The poor outcomes with infected surgical implants are a result of the high antibiotic tolerance of biofilms established on the implant^8,10,11^. It has been well established that traditional antibiotics are unable to eliminate approximately 5–10% of bacterial biofilms^12^. This tolerance is believed to be achieved, in part, through bacterial persisters, a small sub-population of bacteria cells in biofilms, which have a reduced metabolic state^13–16^. This renders the bacteria tolerant to antibiotics, as there is no active metabolic or cell division pathway for the antibiotic to disrupt.

Antimicrobial peptides serve as a potential alternative strategy to traditional antibiotics. Cationic amphipathic peptides (CAPs) selectively bind to bacteria and create pores in both gram-negative and -positive bacterial membranes. Cationic host defense peptides are CAPs that demonstrate the ability to kill bacteria regardless of resistance to antibiotics. However, the use of natural cationic host defense peptides has been limited in the clinic due to suboptimal efficacy and systemic toxicity^17^. Such limitations are indicative of the contextual activity of CAPs, reflective of their evolution as effector molecules of the innate immunity with the ability to prevent infections by specific pathogens interacting with the host in specific environments. As a result, pathophysiological conditions resulting in acidic pH and abnormal salt concentrations may reduce the effectiveness of these CAPs. More importantly, they tend not to work in systemic circulation likely because of the presence of divalent cations and binding of plasma proteins, which restrict their potential use to topical applications. Hence, efforts to develop these CAPs for clinical applications are hampered by the lack of systemic *in vivo* efficacy in animal models.

These limitations motivated the design of synthetic engineered cationic amphipathic peptides (eCAPs), resulting in the extensive characterization of WLBU2 as a lead candidate for potential clinical development. WLBU2 was rationally designed as an idealized helical peptide with optimized amphipathic structure to maximize bacterial membrane selectivity and minimize potential cytotoxicity toward the host^18,19^. To specifically address the limitations of CAPs, we initially demonstrated the broad-spectrum activity of WLBU2 against both gram-positive and -negative bacteria in the presence of saline and divalent cations. With respect to the failure of CAPs to retain activity in systemic circulation, we first developed an *ex-vivo* bacteremia model indicating the potential systemic efficacy of WLBU2. We showed that WLBU2 subsequently displayed efficacy in a murine model of *P. aeruginosa* sepsis. Thus, unlike naturally occurring CAPs, WLBU2 maintains activity under complex biological conditions^20,21^ against common multidrug-resistant (MDR) pathogens, and with minimal toxicity in animal models^22^. WLBU2 has shown activity against planktonic MRSA in addition to a large panel of ESKAPE pathogens^23^. However, despite all these advances compared to overall CAP limitations, the clinical development of WLBU2 would be best justified in the context of the failure of clinically used antibiotics. With the enormous burden of biofilm-associated infections on health care such as medical implants, trauma, and other surgical site infections, more recent studies have been focused on the potential of WLBU2 to either prevent or disrupt bacterial biofilms. Hence, we and others have demonstrated that systemic delivery of WLBU2 is effective against *P. aeruginosa* biofilms associated with cystic fibrosis with minimal toxicity^22,24^, but activity against *S. aureus* antibiotic-resistant biofilms has not been shown. More importantly, this novel functional property has not been demonstrated in a translational model that can further advance the clinical development of WLBU2 as a superior therapeutic option to current antibiotic regimens.

We reasoned that if the activity of eCAP WLBU2 was independent of metabolism, it should be able to eliminate antibiotic tolerant biofilms on surgical implants more effectively than traditional antibiotics. The goal of this study was to determine differences in WLBU2 activity against *S. aureus* in planktonic growth state and in biofilms on surgical implant material as compared to the common clinically used antibiotic, cefazolin, using both *in vitro* and *in vivo* models.

## Materials and Methods

### Bacterial strains and culture

*S. aureus* SH1000^25^ was used for *in vitro* assays and the *in vivo* animal model. In addition, a series of *S. aureus* clinical strains were used for additional *in vitro* biofilm assays (5 methicillin-resistant strains, 4 methicillin-susceptible strains). All strains were inoculated in Tryptic Soy Broth (TSB, Bectin Dickinson and Company) overnight at 37 °C with shaking at 250 rpm. Strains were diluted in Mueller Hinton Broth (MHB; Bectin Dickinson and Company) to a final concentration of 0.5 × 10^6^ CFU/ml using the 0.5 MacFarland Standard (GFS Chemicals) and an Infinite M200 Spectrophotometer (Tecan). All experiments were performed at least in triplicate at three separate times with freshly inoculated cultures. Institutional Review Board guidelines and regulations were followed for the use of clinically derived *S. aureus* strains.

### Minimum inhibitory and bactericidal concentration

The minimum inhibitory concentration (MIC) of cefazolin and WLBU2 for SH1000 in suspension was determined using CLSI assay protocols, incubating freshly plated cultures at 0.5 × 10^6^ CFU/ml for 24 hours with serial dilutions of each antimicrobial and observing inhibition of bacterial growth based on turbidity. Cefazolin concentrations ranged 0.044, 0.088, 0.18, 0.35, and 0.7 µM (0.02, 0.04, 0.08, 0.16, and 0.32 µg/ml). WLBU2 concentrations ranged 0.9, 1.8, 3.7, 7.5, and 15 µM (3.1, 6.2, 12.5, 25, and 50 µg/ml). Both antibiotics were diluted in MHB before addition to SH1000.

The minimum bactericidal concentration (MBC) of cefazolin and WLBU2 for SH1000 in suspension was determined by incubating freshly plated cultures at 0.5 × 10^6^ CFU/ml with antibiotics. Cefazolin concentrations ranged 0.15, 0.35, 0.7, 1.4, and 3.5 μM (0.08, 0.16, 0.32, 0.64, 1.6 µg/ml). WLBU2 concentrations ranged 4.5, 9, 18, 37, 74 μM (15, 31, 62, 125, 250 µg/ml). Well contents were tested at 0, 2, 8, 24, and 48 hours. After treatment, well contents were serial diluted into MHB, and CFU were determined using TSA II with 5% sheep blood CS100 plates that were incubated overnight at 37 °C. The limit of detection was 100 CFU/ml as 10 µl samples of the dilutions were plated.

WLBU2 exhaustion assay was performed by subjecting WLBU2 at 10x MIC (250 µg/ml) to increasing inoculation densities to further assess bactericidal activity. SH1000 plated at 0.1, 1, 10, 100, and 1000 × 10^6^ CFU/ml in suspension for 30 minutes and quantified by serial dilution on blood agar plates.

### *In vitro* biofilm killing assays

Rods were prepared from 0.6 mm diameter stainless steel Kirschner wire (Synthes) and cut into 6 mm length, autoclaved, and plated in wells along with SH1000 and all clinical strains at 1 × 10^6^ CFU/ml. After plating, fresh MHB media was exchanged at 24 hours. At 48 hours, wire with mature biofilms were either placed into fresh media, or treated with either cefazolin at 3.5 μM (1.6 µg/ml) or WLBU2 at 74 µM (250 µg/ml). At 0.5, 1, 6, and 24 hours Kirschner wire were taken from wells, placed into 1% Tween 20 in PBS and sonicated for 10 minutes. Resulting sonication media was serial diluted into MHB and CFU were determined on blood agar plates. Sonicated rods were sterilely placed in fresh MHB for 72 hours and assessed for visual turbidity.

### Persister cell viability assays

SH1000 at 1 × 10^6^ CFU/ml was pre-treated 90 minutes with carbonyl-cyanide-m-chlorophenylhydrazone (CCCP) diluted to 12.5 µg/ml in MHB^16^. Bacterial cultures were centrifuged and pellet re-suspended in MHB before antibiotic treatment. Cefazolin treatment was at 3.5 µM (1.6 µg/ml), WLBU2 was at 74 µM (250 µg/ml). Percent survival was calculated from baseline bacterial cultures after pretreatment but before antibiotic addition. After 6 hours of treatment, serial diluted drop assays were performed on samples and plated on blood agar plates for CFU analysis.

### Viable bacterial biofilm microscopy

SH1000 was plated at 1 × 10^6^ CFU/ml in 8 chambered slides (Lab-Tek), with wells replaced with fresh MHB 24 hours later. After 48 hours biofilms were treated with cefazolin and WLBU2 at 10xMIC (3.5 µM and 74 µM) and placed into 37 °C incubator. After 10 minutes, LIVE/DEAD BacLight Bacterial Viability Kit fluorescent stain (Invitrogen) was added to well contents and incubated at room temperature protected from light for 20 minutes. Fluorescence microscopy performed using a Nikon Eclipse TE2000 microscope with 20x objective, and a Q Imaging RETIGA EXi camera. Images captured and merged using Northern Eclipse software.

### Periprosthetic joint infection animal model

All experiments were performed under approved IACUC animal protocol in University of Pittsburgh Division of Laboratory Animal Resources. Twelve-week-old B57BL/6 J female mice (Jackson) were used for all experiments. Mice where anesthetized by 2% isoflurane, hair was removed from leg and treated with betadine. With a scalpel, a medial parapatellar incision was made, and lateral displacement of the quadriceps-patellar complex allowed for visualization of the femoral intercondylar notch. With a 25-gauge needle, the femoral intramedullary canal was manually reamed. A 0.6 mm wide/6 mm long sterile Kirschner wire (Synthes) was inserted into the canal and was left protruding ~1 mm into the joint. The quadriceps-patellar complex was reduced back to midline and incision was closed using sutures. An inoculation volume of 10 µl with 1 × 10^6^ CFU of SH1000 was injected into the joint space. Treatment group mice received either WLBU2 at 0.01 mg/kg-10 mg/kg, cefazolin at 50 mg/kg, or rifampin at 30 mg/kg) starting 24 hours after surgery and inoculation. Mice received antibiotic dose twice a day for three days. Mice were euthanized and Kirschner wire implant as well as a ~2 mm × 2 mm piece of distal femur were placed in 1% Tween 20 on ice. Implants were sonicated for 10 minutes; distal femur was mechanically homogenized for 30 seconds. Samples were serially diluted into MHB and 10 µl drop assays were performed on blood agar plates.

### Statistics

When comparing two groups, a two tailed Mann-Whitney test was performed, with p < 0.05 considered statistically significant. When comparing multiple groups, a two tailed Kruskal-Wallis test with Dunn’s multiple comparisons post-test was performed, with p < 0.05 considered statistically significant.

## Results

### WLBU2 has high efficacy against planktonic *S. aureus*

**Figure 1:**
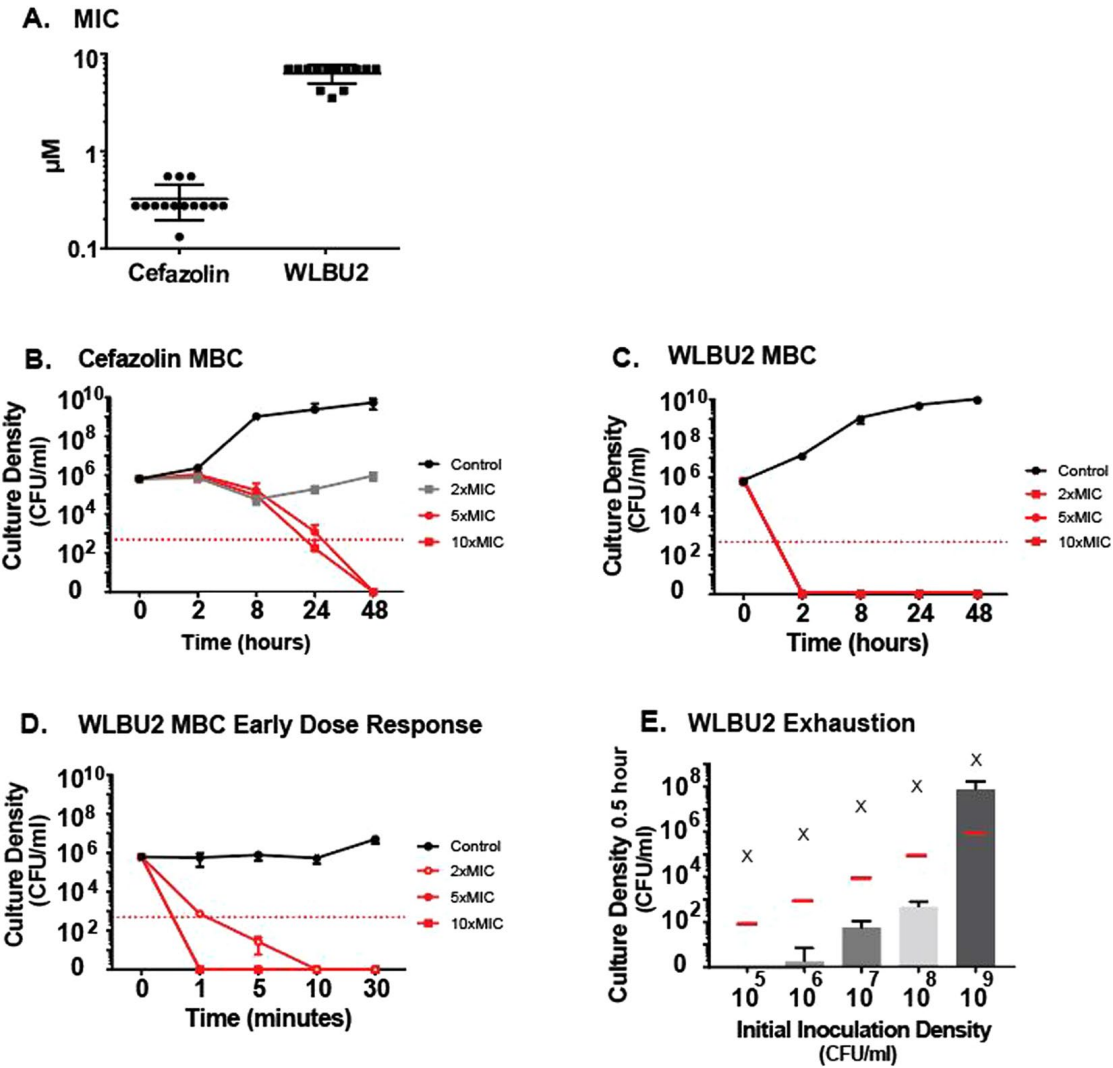
(**A**–**E**) Antimicrobial activity of cefazolin and WLBU2 against planktonic methicillin-sensitive *S. aureus* (SH1000). (**A**) Minimum inhibitory concentration (MIC) of cefazolin and WLBU2 determined by serial dilutions of antibiotics added to SH1000 plated at 0.5 × 10^6^ CFU and overnight culture turbidity. (**B**) Minimum bactericidal concentration (MBC) of cefazolin determined by CFU drop assays at select time points after addition of antibiotic, red dashed line represents 99.9% drop in live bacteria. (**C**) Initial attempt at WLBU2 MBC quantification based on cefazolin temporal progression, antimicrobial peptide yielded sterile conditions. (**D**) CFU analysis on WLBU2 treated samples within 30 minutes after treatment, showing dose response of killing. (**E**) 10xMIC of WLBU2 added to log fold dilutions of SH1000 including overnight stock inoculum (10^9^ CFU/ml) and CFU analysis performed after 30 minutes, WLBU2 at this kills over 99.9% of SH1000 up to 10^8^ CFU/ml.

We first tested the bactericidal effects of WLBU2 with planktonic bacteria. The MIC of cefazolin was found to be 0.37 ± 0.1 µM (0.17 ± 0.05 µg/ml), and that of WLBU2 was 7.85 ± 2.0 µM (26.7 ± 6.7 µg/ml) (Fig. 1A). At a dose of 10xMIC (3.5 μM), cefazolin yielded a three-log reduction of culture after 24 hours (Fig. 1B). Checkerboard assay demonstrated that WLBU2 activity was not augmented by addition of cefazolin. WLBU2 treated cultures to below the limit of detection for CFUs within 2 hours at all WLBU2 concentrations tested (Fig. 1C). To observe WLBU2 dose response, experiments were repeated at shorter time points of less than 60 minutes. A three-log reduction in culture occurred within 1 minute at 10xMIC (74 μM) of WLBU2 (Fig. 1D).

The bactericidal dose response of WLBU2 at increasing bacterial inoculation densities was next evaluated. We wished to determine WLBU2 antimicrobial efficacy against increasing bacterial burden. WLBU2 at 74 µM (250 µg/ml) to overnight cultures diluted to 1 × 10^5^, 1 × 10^6^, 1 × 10^7^, 1 × 10^8^, and 1 × 10^9^ CFU/ml cultures. Quantitative agar culture (CFU assay) was performed after 30 min of exposure to WLBU2, and revealed a three-log reduction in bacterial colony forming units in all groups except 1 × 10^9^ CFU/ml. At 1 × 10^9^ CFU/ml there was a one log reduction in bacterial density (Fig. 1E).

### WLBU2 eliminates *S. aureus* implant biofilms

**Figure 2:**
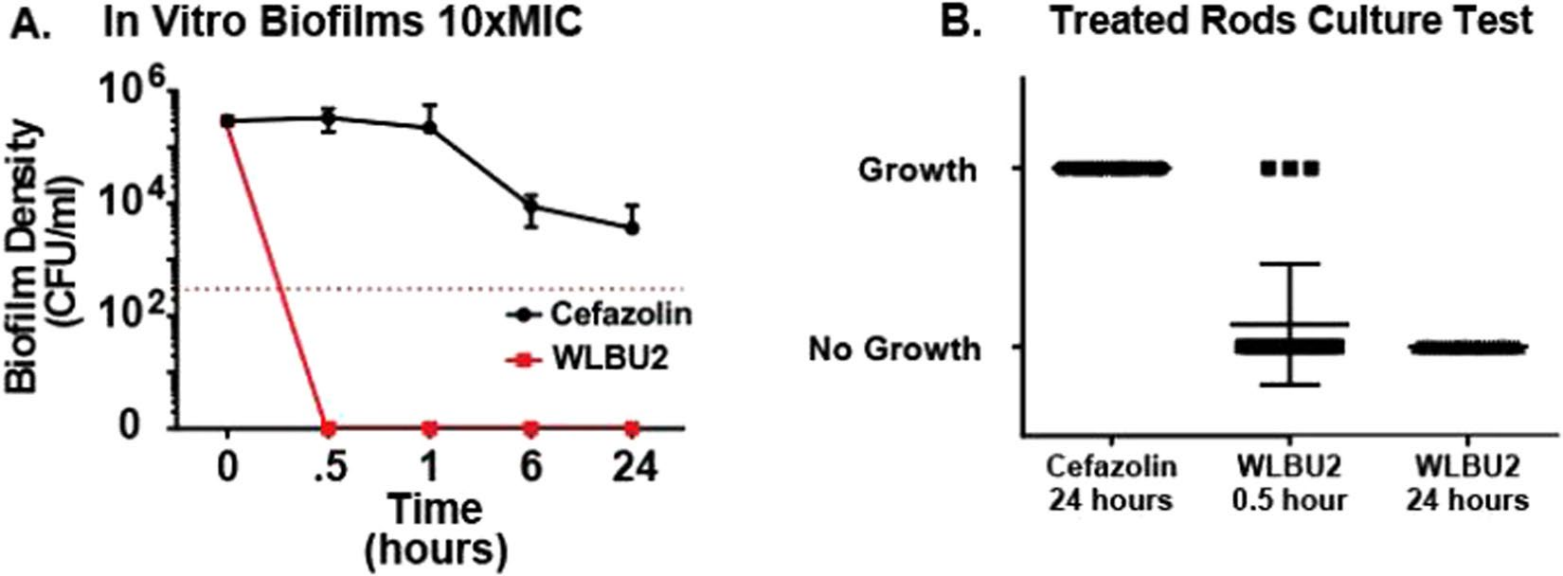
(**A**–**B**) Antimicrobial activity of cefazolin and WLBU2 against *S. aureus* biofilm. (**A**) Mature biofilms grown on Kirschner wire treated with cefazolin or WLBU2 at 10xMIC, CFU analysis shows cefazolin failed to clear 99.9% after 24 hours while WLBU2 sterilized Kirschner wire after 30 minutes, red dashed line represents 99.9% drop in live bacteria compared to pretreatment biofilm CFU (**B**) After CFU assay Kirschner wires placed into fresh MHB and turbidity of culture checked every 24 hours for 3 days, sterile cultures seen in 24 hour WLBU2 treated samples.

Mature SH1000 *S. aureus* biofilms were cultured on stainless steel rods (Kirschner wire; K-wire), and treated with 10xMIC cefazolin and WLBU2. At 24 hours, cefazolin did not achieve a three-log reduction while WLBU2 continued to effectively treat biofilms under the limit of detection after 30 minutes (Fig. 2A). These experiments were repeated with WLBU2 at lower doses of 1, 2.5, and 5xMIC. After 24 hours of treatment, CFU assays showed all WLBU2 treated biofilms were under our limit of detection. To further test for complete elimination of biofilms, implant pieces were sterilely re-cultured with fresh MHB for an additional 72 hours and assessed for turbidity. All of the stainless-steel coupons (24/24; 100%) treated with cefazolin were turbid after 24 hours, whereas only 12.5% (3/24) of the coupons treated with WLBU2 for 0.5 hour were turbid. Strikingly, none of the stainless-steel coupons (0/24; 0%) treated with WLBU2 for 24 hours were turbid, and medium remained clear indicating no viable bacteria were present (Fig. 2B). Clear cultures corresponded with quantitative cultures under our limit of detection. WLBU2 eliminated mature implant biofilms on a model strain of *S. aureus*, SH1000.

We next determined whether WLBU2 would demonstrate similar activity against clinical strains of *S aureus*. Biofilms from clinical strains were treated with cefazolin, vancomycin, and WLBU2 at 10xMIC for 24 hours, and then sterility was tested for 72 hours in fresh media. Strains were composed of 5 methicillin-resistant strains and 4 methicillin-susceptible strains. At 24 hours, WLBU2 treated biofilms all showed culture negative tests. Conversely, cefazolin and vancomycin treated clinical strain biofilms all had 100% and 84% positive cultures after 24 hours (Table 1). WLBU2 eliminated MRSA and MSSA clinical strain surgical implant biofilms comparable to SH1000 biofilms.

**Table 1:** 72-hour culture test from panel of methicillin sensitive and resistant *S. aureus* clinical isolate biofilms after 24 hours of treatment with cefazolin or WLBU2 at 10x MIC. Each strain repeated at least three separate times in triplicate.

Clinical Isolate	Cefazolin Treated	Vancomycin Treated	WLBU2 Treated
MSSA-1	100% (12/12)	100% (10/10)	0% (0/10)
MSSA-2	100% (9/9)	100% (9/9)	0% (0/9)
MSSA-3	100% (9/9)	100% (9/9)	0% (0/9)
MSSA-4	100% (9/9)	25% (3/12)	0% (0/9)
MRSA-1	100% (9/9)	100% (9/9)	0% (0/9)
MRSA-2	100% (9/9)	100% (9/9)	0% (0/9)
MRSA-3	100% (9/9)	50% (5/10)	0% (0/9)
MRSA-4	100% (9/9)	100% (9/9)	0% (0/9)
MRSA-5	100% (9/9)	100% (9/9)	0% (0/9)

### WLBU2 bactericidal action is independent of metabolism and cell division

**Figure 3:**
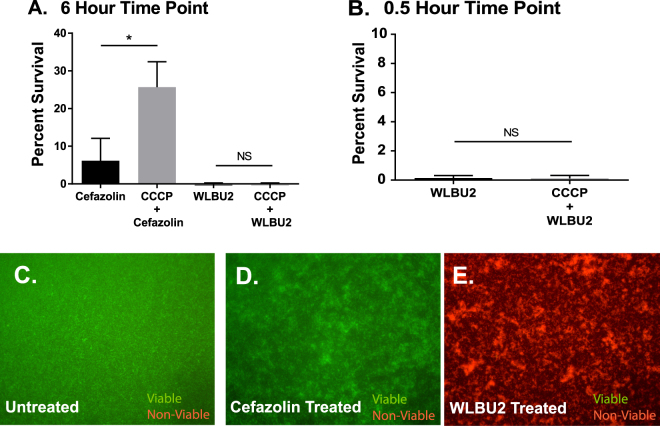
(**A**–**H)** Further evaluation of differences in cefazolin and WLBU2 bactericidal action against SH1000. (**A**) Planktonic SH1000 treated with cefazolin or WLBU2 at 10xMIC after pretreatment with 12.5 μg/ml CCCP, significant increase in percent survival in cefazolin group but not in WLBU2 group. (**B**) CFU assay performed at earlier 30-minute time point after WLBU2 treatment to pretreated and control showed no difference in bactericidal efficacy. Mature biofilms grown on chamber slides were stained with LIVE/DEAD bacterial viability kit and fluorescent microscopy performed after no treatment (**C**) 30-minute cefazolin treatment (**D**), or 30-minute Cefazolin Treated (**E**).

To test if the bactericidal capabilities of WLBU2 were dependent on bacterial metabolism, SH1000 was pre-treated with proton-motive-force disrupting agent carbonylcyanide-m-chlorophenylhydrazone (CCCP) before antibiotic was added to decrease metabolism. Serial dilution assays demonstrated that a dose of 12.5 µg/ml of CCCP for 90 minutes suspended growth of SH1000 for 6 hours with minimal loss of viability. After exposure to cefazolin at 10xMIC (3.5 μM) for 6 hours, SH1000 pre-treated with CCCP had a statistically significant increase in survival compared to cells treated with cefazolin without CCCP with p < 0.0001. Pretreatment with CCCP did not alter the bactericidal ability of WLBU2 as compared to untreated controls (Fig. 3A). Not surprisingly, antimicrobial activity of WLBU2 remained unchanged regardless of metabolic activity even after a 30-minute challenge with p = 0.8867 (Fig. 3B).

The above experiments indicated that bacteria in biofilms were being efficiently killed by WLBU2; however, it was not clear if WLBU2 was also disrupting the biofilms structure. To analyze biofilms structure following WLBU2 challenge and as a second measure of bacterial viability, fluorescent microscopy with LIVE/DEAD staining was performed. Untreated SH1000 biofilms showed mostly viable cells present in the FITC (green) channel with minimal dead cells present in Cy3 (red) channel (Fig. 3C). Treatment of biofilms with cefazolin at 3.5 µM for 30 minutes showed minimal change from untreated biofilms staining and in biofilms structure (Fig. 3D). Treatment of biofilms with WLBU2 at 74 µM for 30 minutes showed a clear drop in viable signal and increase in dead signal and a clear disruption in the biofilms structure (Fig. 3E).

### WLBU2 has comparable efficacy to cefazolin and rifampin in a periprosthetic joint infection murine model

**Figure 4:**
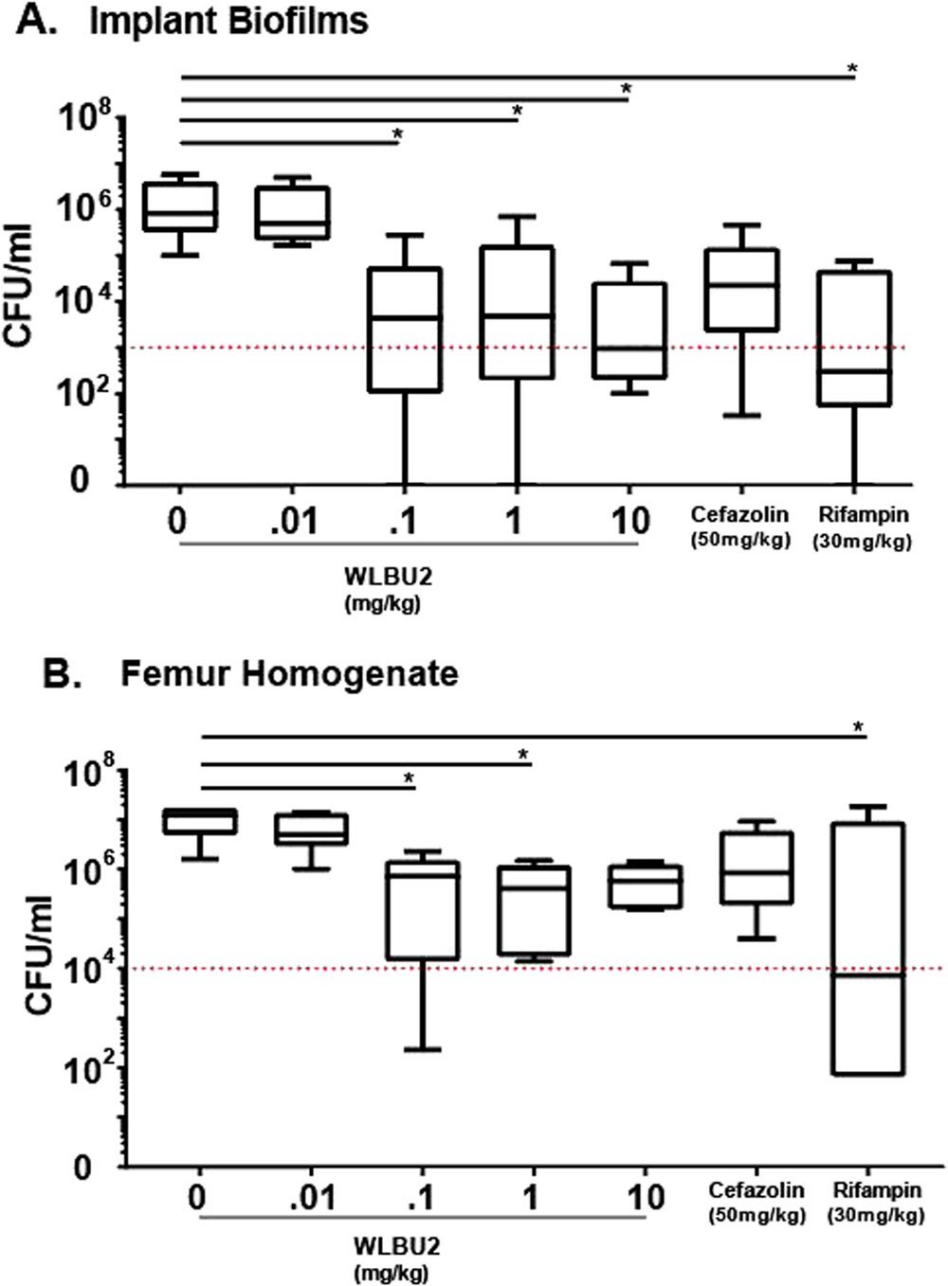
(**A**–**B)** Periprosthetic joint infection (PJI) murine model testing bactericidal efficacy of WLBU2 *in vivo*. Mice received Kirschner wire implant up femoral canal and 1 × 10^6^ CFUs of SH1000 injected into knee joint. Groups received log increases in WLBU2 intraperitoneally twice a day for 3 days and compared to untreated as well as traditional antibiotic treated groups (cefazolin and rifampin). (**A**) Kirscher wire implant placed into 1% Tween 20 and sonicated 10 minutes, drop assays on blood agar plates shows significant reduction of implant biofilm in WLBU2 groups (0.1–10 mg/kg), red dashed line represents 99.9% drop in live bacteria compared to untreated. (**B**) Proximal femur piece cut placed into 1% Tween 20 and homogenized for 60 seconds, shows significant reduction in local bacterial tissue burden in WLBU2 groups, red dashed line represents 99.9% drop in live bacteria compared to untreated.

Periprosthetic joint infection was modeled in a mouse by placing an intra-articular K-wire through the medullary canal of the proximal femur followed by intra-articular inoculation with *S. aureus*. Animals were treated systemically with an intraperitoneal delivery of cefazolin, rifampin, or WLBU2. Intraperitoneal delivery was chosen due to technical difficulties associated with intravenous delivery. Implant sonication and proximal femur homogenate were used to quantify viable bacteria. We observed a dose response for WLBU2 in reduction of biofilm CFU burden with doses between 0.01 and 10 mg/kg. (Fig. 4A). Quantitative agar culture of implant biofilms sonicates showed a statistical significant reduction in 0.1–10 mg/kg WLBU2 treated mice compared to untreated control mice. Cefazolin showed a one log reduction in viable bacteria as compared to untreated controls, but this reduction was not statistically significant. Rifampin had a comparable reduction in biofilms sonicate as compared to WLBU2 (Fig. 4A). Distal femur homogenate of mice showed similar results to paired K-wire implants, with WLBU2 treated samples showing significant reduction compared to untreated controls (Fig. 4B). This was a localized infection model that is not expected to result in life threatening sepsis, therefore no survival study was performed. Due to need for quantification of local tissue around infected implant, histologic analysis of joint was not performed. No significant drop in body weight was observed in mice among treatment groups.

## Discussion

The high tolerance of biofilms to antibiotics makes it difficult to eliminate medical device infections. In total knee arthroplasty, treatment of chronic infection requires removal of the implant followed by an extended course of antibiotics before a new, final implant can be inserted. In this study, we investigated the activity of the eCAP, WLBU2, against *S. aureus* planktonic and biofilm cells as compared to cefazolin. We demonstrate that in the killing of planktonic *S. aureus* cells, WLBU2 and cefazolin had similar activity, but WLBU2 time to kill was approximately three orders of magnitude faster (WLBU2 5 minutes; cefazolin 2 days). Even for notoriously antimicrobial tolerant *S. aureus* biofilms, WLBU2 maintained its activity, disrupted biofilms, and effectively treated biofilms made by strain SH1000 and clinical strains to under the limit of detection. Cefazolin had a large reduction of exhibited bactericidal activity in biofilms as compared to planktonic cells. The mechanism behind WLBU2 activity appeared to be cell lysis and this activity was independent of metabolism. In a periprosthetic joint infection animal model, WLBU2 maintained a superior level of efficacy as compared to cefazolin and no obvious toxicity.

WLBU2 maintained comparable activity between *S. aureus* planktonic and biofilm cells. This is in sharp contrast to other antibiotics where there is a large loss of activity between biofilms as compared to planktonic cells^12,26–28^. WLBU2 maintained its bactericidal action against SH1000 biofilms, as well as established biofilms of MSSA and MRSA clinical strains after less than 1 hour. There have been few other antimicrobial chemotherapeutic agents that have demonstrated an ability to eliminate persistent biofilms. A novel antibiotic, ADEP, can activate the bacterial protease, ClpP, independent of ATP in bacterial persisters, inducing metabolic activity and allowing for total clearance of infection in combination with a traditional antibiotic, rifampin^29^. Optimal ADEP activity requires the addition of a secondary antibiotic, rifampin. A second approach includes using a chemotherapeutic agents, Mitomycin C and cisplatin. Cisplatin is found to eradicate persister cells in clinical strains of *S. aureus* ^30^. It is unclear if the dosing necessary for these chemotherapeutic agents to eradicate biofilms falls within the range of systemic toxicity associated with systemic dosing for oncologic disease.

Bacterial metabolism had no effect on WLBU2 activity. The decreased metabolic activity of bacterial persister cells has been proposed as a mechanism behind biofilms antibiotic tolerance. Chronic infections are facilitated by the survival of dormant persister cells^31^. In *S. aureus*, metabolically dormant stationary bacteria with depleted ATP levels are associated with wide-spectrum antibiotic tolerance^32^. Based on these findings, we reasoned that if the antimicrobial peptide WLBU2 could eliminate *S. aureus* biofilms, then the mechanism was likely independent of metabolic state of the bacterial cells. When CCCP, a chemical inhibitor of oxidative phosphorylation and proton-motive force, was used to decrease metabolic activity in *S. aureus* ^16^, WLBU2 activity was unchanged as compared to a statistically significant decrease in the activity of cefazolin.

Host defense peptides have two possible limitations that include systemic toxicity and labile activity related to proteases, pH, and ionic strength. These major limitations were tested in the periprosthetic joint infection animal model. Our group did not observe systemic toxicity with WLBU2 in the initial range of therapeutic efficacy. This agrees with previously published data that demonstrate minimal eukaryotic cytotoxicity *in vitro* and *in vivo* ^18–22^. Further, WLBU2 had greater efficacy compared to cefazolin with systemic delivery demonstrating the ability to maintain a stable level of activity. Other groups have demonstrated that WLBU2 maintains its activity under diverse physiologic conditions, which supports our results^19–22^. Although WLBU2 demonstrated a high level of efficacy in our animal model, the effect was not as robust as inferred from the *in vitro* results. This attenuation in efficacy suggests that WLBU2 is still inhibited to a limited degree by these or other factors not accounted for in our *in vitro* studies. Changes in bactericidal action of antimicrobial peptides have been shown in other animal models, with peptide bioavailability reduced due to protease activity *in vivo* ^17^. This shortcoming can be overcome by carefully designed D-enatiomers of WLBU2 as shown by previous studies of other cationic peptides^33,34^.

*S. aureus* biofilms are highly tolerant of traditional antibiotics making surgical implant infections an enormous clinical challenge. Our data add to growing evidence that the eCAP WLBU2 has high efficacy *in vitro* and *in vivo* with minimal systemic toxicity. WLBU2 could eliminate *S. aureus* biofilms regardless of their methicillin resistance status, which has not been demonstrated by other clinically available chemotherapeutic agents. CCCP treatment experimental results were consistent with WLBU2 activity being independent of bacterial metabolism and cell division, which has not been previously demonstrated with other antimicrobial peptides. WLBU2 shows promise as a novel therapeutic in the treatment of *S. aureus* infections in the challenging setting of surgical implants such as periprosthetic joint infection. Considering the previously demonstrated antibacterial activity of WLBU2 against diverse multidrug-resistant bacterial strains^23^, WLBU2 may offer a novel effective treatment for periprosthetic joint infections involving antibiotic-resistant bacterial biofilms, including those associated with knee replacement surgeries.
